# Serum Thyrotropin Levels Across Older Age Groups in Geriatric Outpatients Without Documented or Clinically Evident Thyroid Disease: A Retrospective Cross-Sectional Study

**DOI:** 10.3390/jcm15208059

**Published:** 2026-10-17

**Authors:** Sibel Akbaş, Ayşegül Uçkun, Oğuzcan Gümüşçubuk, Ahmet Yalçın, Murat Varlı, Volkan Atmış

**Affiliations:** 1 Division of Geriatrics, Department of Internal Medicine, Kocaeli City Hospital, 41060 İzmit, Türkiye; 2 Department of Medical Oncology, Dr. Abdurrahman Yurtaslan Ankara Oncology Training and Research Hospital, University of Health Sciences, 06200 Ankara, Türkiye; 3 Department of Geriatric Medicine, Sincan Training and Research Hospital, 06949 Ankara, Türkiye; 4 Division of Geriatrics, Department of Internal Medicine, Faculty of Medicine, Ankara University, 06230 Ankara, Türkiye

**Keywords:** thyroid-stimulating hormone, thyrotropin, aging, older adults, geriatrics, sex differences

## Abstract

**Background/Objectives:** Reported age patterns in serum thyroid-stimulating hormone (TSH) vary across populations and assays. We examined age and sex associations with TSH levels in clinically selected geriatric outpatients in Ankara, Türkiye. **Methods:** This retrospective cross-sectional study analyzed records from 1 October to 31 December 2022. After clinical, medication, and laboratory exclusions, 206 participants aged ≥65 years remained. Linear regression of log-transformed TSH included age per five years and sex, with HC3 robust standard errors. Sensitivity models added comorbidities and medication count and applied the institution-specific TSH and fT4 reference intervals. Exploratory quantile regression used bootstrap confidence intervals (CIs) and formal comparisons of percentile-specific coefficients. **Results:** Mean age was 73.9 ± 6.5 years; 122 participants (59.2%) were women. Median TSH was 1.80 mIU/L (interquartile range, 1.25–2.61) and did not differ across age groups (*p* = 0.151). The primary age estimate was −6.6% per five years (95% CI, −13.1% to +0.5%; *p* = 0.066) and was unchanged after comorbidity adjustment (−6.7%; *p* = 0.077), but attenuated when analysis was restricted to the institutional TSH interval (−3.2%; *p* = 0.274). Women had 25.5% higher TSH in the primary model (95% CI, +1.3% to +55.5%; *p* = 0.037). An exploratory 10th-percentile age estimate (−19.6%; 95% bootstrap CI, −37.4% to −6.6%) was not supported by a joint test of percentile differences (*p* = 0.13). **Conclusions:** No statistically significant age association was demonstrated in this selected cohort. The primary analysis indicated higher TSH in women, although precision varied across restricted analyses. Isolated TSH results in older outpatients should be confirmed and interpreted in a clinical context; larger prospective studies are needed.

## 1. Introduction

Serum thyroid-stimulating hormone (TSH) is central to the evaluation of primary thyroid dysfunction, yet its interpretation in later life is not straightforward. Age-related changes in hypothalamic–pituitary–thyroid set points, comorbidity, medication exposure, non-thyroidal illness, autoimmunity, and iodine status may all alter population distributions of TSH [1,2,3]. Misclassification matters clinically: modest elevations can prompt repeat testing, diagnostic labeling, and treatment, while excessive treatment in older adults may increase cardiovascular and skeletal risk [2,3].

Large cross-sectional datasets have frequently shown an upward shift in the TSH distribution with age [4,5], and longitudinal data have also described within-person increases [6]. However, the magnitude and direction of age effects vary with reference-population selection, sex, ethnicity, assay, antibody status, and iodine exposure [7,8,9,10]. An indirect analysis in older adults in Eastern Türkiye reported no consistent age-related pattern in TSH and higher TSH in women [11]. Turkish laboratory and community studies further show that locally observed thyroid indices depend on study design, assay, and iodine context [12,13]. Clinically characterized, age-specific geriatric evidence from Türkiye therefore remains limited, including across regions with differing iodine exposure.

We therefore examined serum TSH in a clinically selected cohort of geriatric outpatients with no documented or detected thyroid disease after evaluation of medical history, physical examination findings, medications, and available laboratory data at a tertiary center in Ankara. The primary objective was to estimate the association between continuous age and TSH after accounting for sex. Secondary objectives were to compare TSH across conventional geriatric age groups and to describe sex-related differences. We explicitly did not attempt to establish a population reference interval.

## 2. Materials and Methods

### 2.1. Study Design and Setting

We conducted a single-center retrospective cross-sectional study in the geriatric medicine outpatient clinic at Ankara University Faculty of Medicine, Ankara, Türkiye. Electronic records of visits between 1 October and 31 December 2022 were reviewed between 1 March and 1 April 2023, after ethics approval had been obtained. Reporting was guided by the Strengthening the Reporting of Observational Studies in Epidemiology (STROBE) statement [14].

### 2.2. Participants

The clinical screening log documented 477 outpatient encounters. Twenty-one repeat encounters were removed, leaving 456 unique patients for eligibility assessment. Eligibility was evaluated using the documented medical history and physical examination, medication list, and available laboratory results. Patients were excluded for documented hypothyroidism, hyperthyroidism, or thyroid cancer; levothyroxine use; previous thyroid surgery or radioactive iodine treatment; medications known to affect thyroid function (including amiodarone, glucocorticoids, lithium, levodopa, phenobarbital, phenytoin, carbamazepine, propranolol, propylthiouracil, tyrosine-kinase inhibitors, interferon, interleukin-2, octreotide, dopamine, or dobutamine); active malignancy; or acute infection or inflammatory disease. Acute illness, including infection and acute flares of malignant or rheumatological disease, was ruled out based on history, physical examination, and laboratory tests; patients with an erythrocyte sedimentation rate ≥ 30 mm/h, C-reactive protein ≥ 5 mg/L, and/or a leukocyte count ≥ 11,000/µL were excluded. For patients with malignancy followed at our institution’s oncology department, remission status was verified in the hospital information system, and patients not in remission were excluded. For patients with malignancy in remission followed elsewhere, and for patients with only one of the two markers (erythrocyte sedimentation rate or C-reactive protein) available, fT3 and fT4 were required to exclude non-thyroidal illness, and patients without these results were excluded. Thyroid cancer, thyroid surgery, radioactive iodine treatment, levothyroxine, and other medications were verified from the outpatient history recorded in the hospital information system.

**Figure 1:**
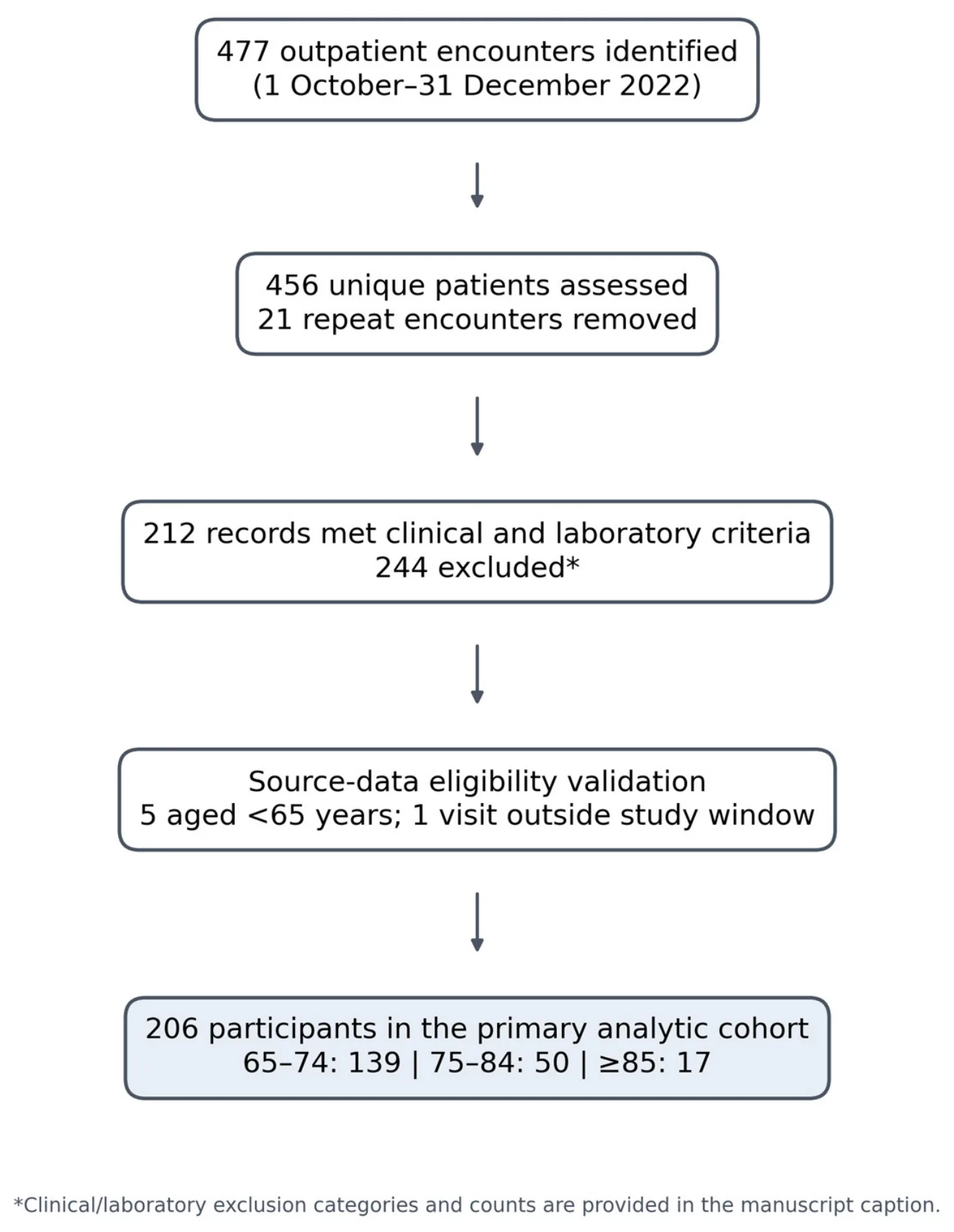
Participant flow. Clinical/laboratory exclusions totaled 244: documented thyroid disease or treatment (*n* = 83), active malignancy or insufficient information to exclude non-thyroidal illness in malignancy follow-up (*n* = 10), missing inflammatory markers or insufficient thyroid tests in specified cases (*n* = 32), elevated C-reactive protein (*n* = 87), elevated erythrocyte sedimentation rate (*n* = 12), leukocytosis (*n* = 13), thyroid-active medication use (*n* = 6), and unknown medications (*n* = 1).

After these clinical and laboratory exclusions, 212 records remained. A source-data validation step recalculated age from date of birth and visit date and verified the study window. Five records were from patients aged <65 years at the visit, and one visit occurred on 30 September 2022; these six records were excluded. The final analytic cohort comprised 206 participants (Figure 1).

### 2.3. Variables and Laboratory Assessment

Age was calculated from date of birth and visit date and analyzed continuously and in three categories: 65–74, 75–84, and ≥85 years. Sex was recorded as female or male in the electronic health record; gender identity was unavailable. Comorbidities were abstracted from the record. Serum TSH was routinely requested in the geriatric clinic and measured in the Ankara University Faculty of Medicine Central Laboratory using the Elecsys TSH electrochemiluminescence immunoassay (ECLIA), with manufacturer-supplied reagents, on the cobas 8000 modular analyzer platform (Roche Diagnostics GmbH, Mannheim, Germany). The laboratory reference interval used during the study period was 0.27–4.20 mIU/L. According to the clinic protocol, free triiodothyronine (fT3) and free thyroxine (fT4) were generally requested when TSH was outside the laboratory interval to identify overt thyroid dysfunction and were required to exclude non-thyroidal illness in patients with malignancy in remission who were followed outside the institution and in patients for whom only either C-reactive protein or erythrocyte sedimentation rate was available; otherwise, they were requested at the treating physician’s discretion. Because testing was conditional on clinical indication, fT3 and fT4 were reported descriptively and were not included in inferential analyses.

The laboratory reference interval for fT4 during the study period was 12–22 pmol/L. Laboratory sampling dates were recorded without clock time. One erroneous year entry in the laboratory-date field was corrected against the source medical record before the timing counts were calculated. The blood sample was obtained on the day of the index visit in 187 of 205 participants with a recorded sampling date (91.2%) and within seven days of the visit in 193 (94.1%); the remaining samples were dated between 57 days before and 7 days after the visit. Blood samples were drawn in the morning after an overnight fast as part of routine outpatient practice; the clock time of sampling and specimen storage conditions were not recorded in the electronic record. All measurements were performed in the same central laboratory on a single analyzer platform; run-level quality-control records were not retrievable retrospectively.

Documented thyroid disease and treatment history were determined from outpatient histories and medication records. For the single participant whose structured medication field was recorded as unknown, source-chart review confirmed that no medication known to affect thyroid function was being used. Values outside the institutional TSH interval were retained in the primary analysis because the absence of a documented diagnosis was the clinical eligibility criterion; sensitivity analyses restricted TSH to 0.27–4.20 mIU/L and jointly restricted TSH and fT4 to their institutional intervals.

### 2.4. Outcomes

The primary outcome was the relative difference in TSH associated with each five-year increase in age after adjustment for sex. Secondary outcomes were the rank-based association between age and TSH, differences in TSH among the three age groups, and the association between sex and TSH. Associations at the 10th, 50th, and 90th conditional percentiles were examined post hoc and were considered exploratory. Estimation of reference limits for clinical use was not an objective; accordingly, CLSI reference-interval procedures were not applied [15].

### 2.5. Statistical Analysis

Continuous variables are reported as mean ± standard deviation or median and interquartile range (IQR), as appropriate; categorical variables are reported as *n* (%). TSH was right-skewed and was log-transformed for regression. The primary model was ordinary least-squares regression of log(TSH) on age per five years and sex, with heteroscedasticity-consistent (HC3) standard errors and t-based inference. Coefficients were exponentiated and reported as percentage differences with 95% CIs. Spearman correlation assessed the unadjusted continuous association, the Kruskal–Wallis test compared age groups, and the Mann–Whitney U test compared sexes. The primary sensitivity analysis was restricted to TSH values within the institutional interval (0.27–4.20 mIU/L).

In response to peer review, additional post hoc sensitivity models were fitted: (i) adjustment for the comorbidities recorded in Table 1 (hypertension, diabetes mellitus, coronary artery disease, chronic kidney disease, and dementia), with and without the number of regularly used medications abstracted from the visit record; (ii) separate exclusion of participants with TSH < 0.27 mIU/L or >4.20 mIU/L; (iii) restriction to participants with both TSH and fT4 within the institutional intervals; (iv) sex-stratified models and an age-by-sex interaction; and (v) an eGFR-adjusted model, reported only in the Supplementary Materials because creatinine-based eGFR equations include age and may therefore overadjust the age association. Variance inflation factors were examined in the comorbidity model.

Exploratory post hoc quantile regressions of log(TSH) on age per five years and sex were fitted at the 10th, 25th, 50th, 75th, and 90th percentiles; the 10th, 50th, and 90th percentiles were the originally reported percentiles, and the 25th and 75th were added for graphical display. Their 95% percentile CIs were obtained by case bootstrapping with 5000 resamples (seed 20260829). Differences between the age and sex coefficients at the 10th, 50th, and 90th percentiles were evaluated using paired bootstrap distributions and a joint Wald test based on the bootstrap covariance matrix. Lower-tail stability was examined by leave-one-out analysis and by excluding participants with TSH < 0.27 mIU/L. These exploratory tests were not adjusted for multiplicity.

Missing values were not imputed; models used complete cases, and the number of participants contributing to each model is reported (Supplementary Table S1). All tests were two-sided. The parent thesis protocol used a prevalence-based sample-size calculation that targeted at least 107 participants; because that calculation addressed thyroid-dysfunction prevalence rather than the continuous age–TSH association reported here, all eligible records in the predefined window were included, and precision is conveyed by CIs and by the minimum effect detectable with 80% power at a two-sided α of 0.05, calculated from the standard errors of the fitted models. Statistical analyses were performed using IBM SPSS Statistics for Windows, Version 27.0 (IBM Corp., Armonk, NY, USA), and R version 4.4.1 (R Foundation for Statistical Computing, Vienna, Austria).

### 2.6. Ethics

The study was approved by the Ankara University Human Research Ethics Committee on 22 February 2023 (decision no. İ02-86-23) and was conducted in accordance with the Declaration of Helsinki. Given the retrospective, non-interventional design of the study, which involved a review of existing medical records without direct contact with participants or any study-specific procedures, study-specific informed consent was not obtained. No information that could identify participants is included in this article.

## 3. Results

### 3.1. Participant Flow and Characteristics

Of the 477 encounters, 206 participants met the clinical, laboratory, age, and visit-window criteria (Figure 1). The mean age was 73.9 ± 6.5 years, and 122 participants (59.2%) were women. The 65–74, 75–84, and ≥85-year groups included 139 (67.5%), 50 (24.3%), and 17 (8.3%) participants, respectively. Hypertension was the most common recorded comorbidity (137/205, 66.8%), followed by diabetes (82/205, 40.0%) (Table 1).

Comorbidity fields were missing for one participant (hypertension, diabetes, coronary artery disease, and chronic kidney disease) and for three participants (dementia); fT4 and fT3 were unavailable in 44 (21.4%) and 122 (59.2%) participants, respectively, because they were requested selectively (Supplementary Table S1). Participants used a median of 3.5 (IQR, 2.0–5.0) regular medications, and 76 (36.9%) used five or more.

### 3.2. TSH Distribution by Age Group and Sex

**Figure 2:**
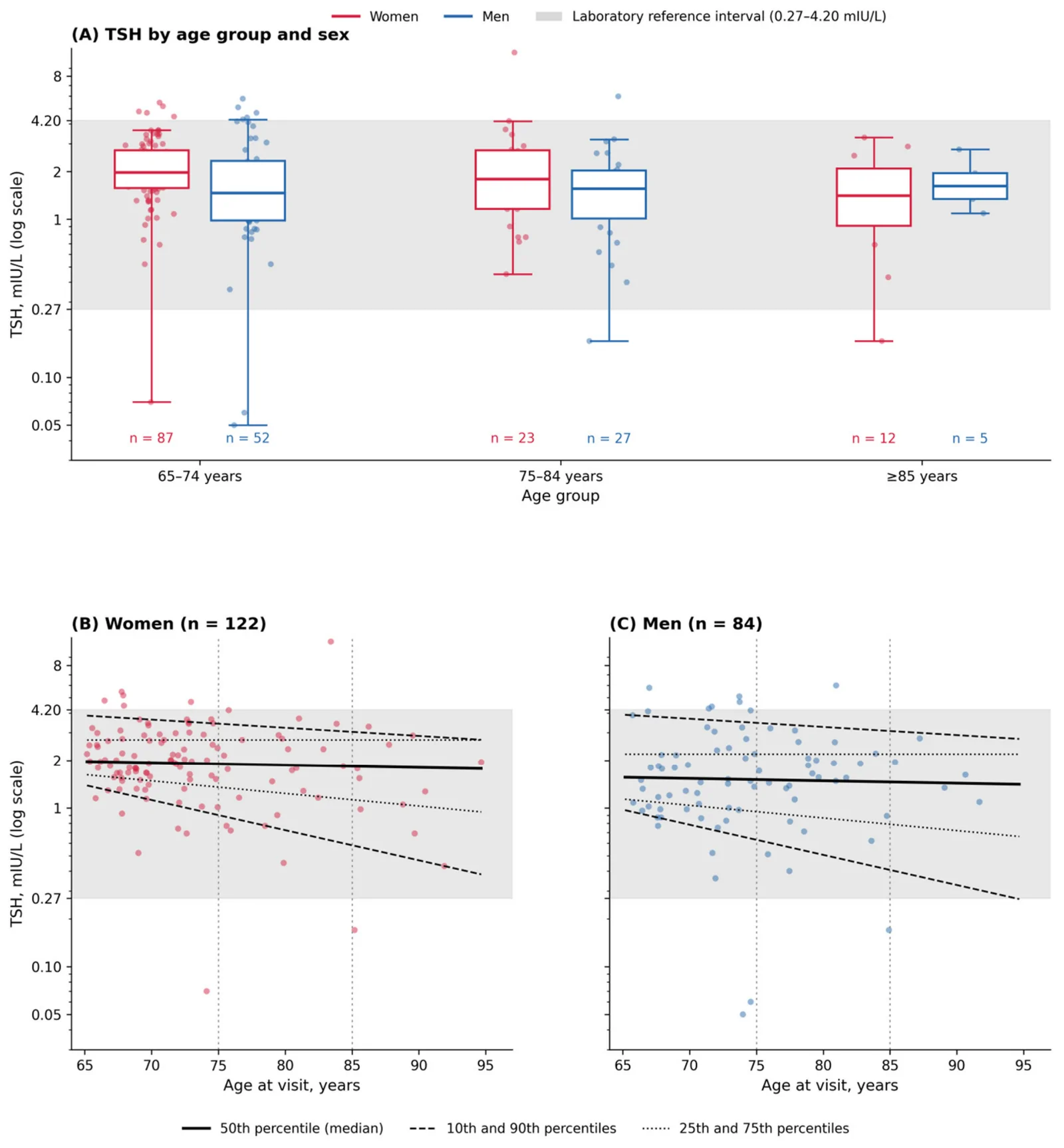
Serum TSH by age and sex in the analytic cohort (*n* = 206): (**A**) TSH by age group and sex; boxes show medians and interquartile ranges, whiskers extend to the most extreme values within 1.5 × IQR of the box, and points show individual participants (horizontal jitter added for visibility). (**B**,**C**) Individual TSH values plotted against age at the visit in women (**B**) and men (**C**), with fitted conditional 10th, 25th, 50th, 75th, and 90th percentiles from the exploratory sex-adjusted quantile regressions; the lines for women and men are parallel on the logarithmic scale because the models contained no age-by-sex term. Vertical dotted lines mark the age-group boundaries (75 and 85 years), and the shaded band is the institutional laboratory reference interval (0.27–4.20 mIU/L). TSH is plotted on a logarithmic scale. IQR, interquartile range; TSH, thyroid-stimulating hormone.

Median TSH in the full cohort was 1.80 mIU/L (IQR, 1.25–2.61). Five participants (2.4%) had TSH < 0.27 mIU/L and 12 (5.8%) had TSH > 4.20 mIU/L. Across ascending age categories (65–74, 75–84, and ≥85 years), median TSH values were 1.84, 1.73, and 1.54 mIU/L, respectively; the overall difference was not statistically significant (Kruskal–Wallis H = 3.78, *p* = 0.151). The inverse correlation between age and TSH was also not statistically significant (Spearman ρ = −0.106, *p* = 0.130). Women had a higher median TSH than men (1.92 [IQR, 1.47–2.70] vs. 1.51 [IQR, 1.00–2.20] mIU/L; Mann–Whitney *p* = 0.010). Within women, median TSH was 1.97, 1.79, and 1.41 mIU/L in the three ascending age groups (*n* = 87, 23, and 12), whereas within men it was 1.46, 1.56, and 1.62 mIU/L (*n* = 52, 27, and 5) (Table 1; Figure 2A). fT4 was available in 162 participants (78.6%), including 15 of the 17 participants whose TSH lay outside the institutional interval; fT3 was available in 84 (40.8%), including 7 of those 17. Median fT4 was 15.3 pmol/L (IQR, 14.0–16.5) overall and 14.3 pmol/L (IQR, 13.2–16.6; range, 11.3–18.7) among participants with out-of-range TSH. Individual values for the 17 participants with out-of-range TSH are listed in Supplementary Table S3. fT4 was below its institutional interval in three of the 15 tested participants (with TSH values of 0.07, 5.97, and 11.30 mIU/L) and was elevated in none; fT4 was unavailable in one participant with TSH < 0.27 mIU/L and one with TSH > 4.20 mIU/L.

### 3.3. Regression and Sensitivity Analyses

In the sex-adjusted primary model, each five-year increase in age was associated with a 6.6% lower TSH (95% CI, −13.1% to +0.5%; *p* = 0.066). Female sex was associated with 25.5% higher TSH (95% CI, +1.3% to +55.5%; *p* = 0.037). In the sex-adjusted age-group model, TSH was 6.4% lower in the 75–84-year group (95% CI, −26.6% to +19.4%; *p* = 0.594) and 25.9% lower in the ≥85-year group (95% CI, −50.4% to +10.5%; *p* = 0.140) than in the 65–74-year group (Table 2). When analysis was restricted to 189 participants with TSH within 0.27–4.20 mIU/L, the age estimate attenuated to −3.2% per five years (95% CI, −8.6% to +2.6%; *p* = 0.274), while the sex estimate remained positive (+23.2% in women; 95% CI, +5.7% to +43.6%; *p* = 0.008).

Additional adjustment for hypertension, diabetes mellitus, coronary artery disease, chronic kidney disease, and dementia left the estimates essentially unchanged (age, −6.7% per five years; 95% CI, −13.5% to +0.7%; *p* = 0.077; women, +29.6%; 95% CI, +4.2% to +61.1%; *p* = 0.020; *n* = 203), as did further adjustment for the number of regular medications (age, −6.5%; 95% CI, −13.4% to +1.0%; *p* = 0.086; women, +29.1%; 95% CI, +3.9% to +60.6%; *p* = 0.022); none of the comorbidity terms was associated with TSH (Supplementary Table S2). The age estimate attenuated after excluding the five participants with TSH < 0.27 mIU/L (−3.9%; 95% CI, −9.8% to +2.4%; *p* = 0.221; *n* = 201) and in the 142 participants with both TSH and fT4 within the institutional intervals (−3.0%; 95% CI, −10.3% to +4.9%; *p* = 0.445). Excluding the 12 participants with TSH > 4.20 mIU/L produced an age estimate of −5.8% (95% CI, −12.1% to +0.8%; *p* = 0.084; *n* = 194). The point estimate for female sex was positive in every model, including after exclusion of participants with TSH < 0.27 mIU/L (+18.9%; 95% CI, +0.6% to +40.6%; *p* = 0.043), exclusion of participants with TSH > 4.20 mIU/L (+30.6%; 95% CI, +6.1% to +60.7%; *p* = 0.012), and restriction to both institutional intervals (+37.4%; 95% CI, +15.1% to +64.0%; *p* = 0.001). Sex-stratified age estimates were −8.7% per five years in women (95% CI, −17.0% to +0.3%; *p* = 0.059) and −2.7% in men (95% CI, −13.4% to +9.3%; *p* = 0.643); the age-by-sex interaction was not statistically significant (*p* = 0.397) (Table 2).

The standard error of the primary age coefficient implies that the study had 80% power to detect a change of approximately 10% in TSH per five years of age (a 9.8% decrease or a 10.9% increase); for the comparison of the ≥85-year group with the 65–74-year group, only a decrease of about 43% or an increase of about 77% was detectable with 80% power. The observed estimates lie within these limits, so the non-significant results should be read as imprecise rather than as evidence of no association.

### 3.4. Distribution of TSH Across Percentiles

In exploratory post hoc quantile regression, the age estimate was −5.8% per five years at the 90th percentile (95% bootstrap CI, −13.9% to +4.7%), −1.7% at the median (95% bootstrap CI, −8.4% to +7.2%), and −19.6% at the 10th percentile (95% bootstrap CI, −37.4% to −6.6%) (Table 3; Figure 2B,C). Estimates for female versus male sex were +43.1% at the 10th percentile (95% bootstrap CI, +2.9% to +123.2%), +26.0% at the median (95% bootstrap CI, +1.3% to +49.4%), and −1.2% at the 90th percentile (95% bootstrap CI, −25.7% to +29.4%). The bootstrap standard error of the age coefficient at the 10th percentile (0.107 on the log scale) was approximately three times that at the median (0.036), reflecting sparse information in the lower tail: 22 observations lay at or below the fitted 10th-percentile line. Percentile-specific age coefficients did not differ significantly in a joint test across the 10th, 50th, and 90th percentiles (Wald χ^2^ = 4.09, df = 2, *p* = 0.13). In pairwise comparisons, the paired-bootstrap CI for the difference between the 10th- and 50th-percentile age coefficients excluded zero (−0.20 log units; 95% CI, −0.45 to −0.05; bootstrap *p* = 0.006), but a normal-approximation test using the bootstrap standard error did not reach significance (*p* = 0.057), and the 10th-versus-90th-percentile difference was not significant (−0.16; 95% CI, −0.44 to +0.01; bootstrap *p* = 0.076). Sex coefficients did not differ significantly across percentiles (joint *p* = 0.21). The 10th-percentile age estimate was insensitive to deletion of any single participant (leave-one-out range, −21.7% to −19.6%) but attenuated to −11.8% per five years (95% bootstrap CI, −21.9% to −2.0%) after exclusion of the five participants with TSH < 0.27 mIU/L. None of these intervals was adjusted for multiplicity.

Descriptively, the proportion of participants with TSH < 1.0 mIU/L was 13.7% (19/139) at ages 65–74 years, 24.0% (12/50) at ages 75–84 years, and 23.5% (4/17) at ages ≥ 85 years. Twenty-two participants had TSH at or below the empirical 10th-percentile threshold of 0.77 mIU/L. fT4 was available for 17 of these 22 participants and had a median of 14.4 pmol/L (IQR, 14.0–17.9). Because fT4 was selectively requested and was unavailable for five participants in this subgroup, overt thyroid dysfunction cannot be excluded for every low-TSH participant.

## 4. Discussion

### 4.1. Principal Findings

In this clinically screened geriatric outpatient cohort, TSH did not differ significantly among conventional older age groups, and the continuous age association in the primary model did not reach statistical significance. The point estimate suggested lower rather than higher TSH with advancing age; it was unchanged by adjustment for recorded comorbidities and medication count but attenuated after restriction to the institutional interval and after exclusion of participants with TSH below 0.27 mIU/L. Exploratory quantile models did not demonstrate an upward age association at the 90th percentile and suggested a negative association at the 10th percentile; however, percentile-specific coefficients did not differ significantly in a joint test, the lower-tail estimate was imprecise, and it attenuated after removal of participants below the institutional lower limit. The primary model indicated higher TSH in women, and female-sex point estimates remained positive across sensitivity models, although precision varied. Taken together, the data are compatible with a sex difference in the TSH distribution within this selected cohort and provide no evidence of an age-related increase; they do not establish an age-related decrease.

### 4.2. Comparison with Previous Evidence

Our age finding differs from the upward age shift reported in NHANES III and other large population studies [4,5,6]. Such comparisons require caution because those studies used different sampling frames, assays, antibody criteria, and population structures. A systematic review identified age, sex, iodine status, ethnicity, assay, and reference-population definition as important sources of variation in TSH limits [7]. More recent large analyses similarly support heterogeneity rather than a universal age trajectory [8,9,10].

Our findings are directionally compatible with the 2026 Eastern Türkiye study, in which TSH did not show a consistent age pattern among older adults and was higher in women [11]. That study used an indirect laboratory-database approach and derived a TSH interval of 0.41–3.94 mIU/L; our cohort adds participant-level clinical screening based on history, examination, medications, and laboratory findings rather than a second indirect estimate. A contemporaneous multicenter indirect study of routine data from three Ankara hospitals derived age- and sex-specific thyroid reference intervals across pediatric and adult age strata [16]. Because that study used a different assay platform and did not clinically adjudicate older participants, our clinically screened geriatric cohort addresses a distinct and complementary question. The higher TSH observed in women is also consistent with contemporary analyses identifying sex as a determinant of thyroid-test distributions [7,8,11,16].

Iodine exposure may be particularly relevant in Ankara. A community study conducted in older residents of Mamak reported a mean urinary iodine concentration of 98 µg/L and concluded that iodine status had not reached favorable levels after salt iodization [13]. However, that study predated the present cohort by more than a decade, and we did not measure urinary iodine; iodine status therefore remains a hypothesis rather than an explanation for our results.

### 4.3. Clinical Interpretation

The clinical implication of these findings is not a new numerical cutoff but a more individualized interpretation of TSH in older adults. Treatment should not be initiated or changed on the basis of a single TSH result alone; repeat measurement, free thyroid hormones, symptoms, comorbidities, medication exposure, and the degree and persistence of the abnormality should be considered together [2,3]. When an earlier stable TSH value is available, within-person change may provide useful context for a threshold crossing. Seventeen participants had TSH outside the institutional interval despite no documented or clinically evident thyroid disease, illustrating the practical value of confirmation and clinical reassessment. Studies in the oldest old likewise show that the clinical meaning of modest TSH variation may differ from that in younger adults [17]. The sex difference observed here describes the distribution of TSH within a selected clinical cohort. It does not by itself establish that sex-specific decision thresholds would improve classification or outcomes; that question requires appropriately selected reference populations with outcome data [7,8,16].

### 4.4. The Lower End of the TSH Distribution

The strongest exploratory age-related estimate occurred at the lower end of the distribution. The modeled 10th percentile was negatively associated with age, and the proportion with TSH < 1.0 mIU/L was higher in the two older groups than at ages 65–74 years. Several features argue for caution. The 10th-percentile estimate rested on 22 observations at or below the fitted line and was about three times less precise than the median estimate; its difference from the median and 90th-percentile coefficients was not significant in a joint test and depended on the testing method; and it attenuated from −19.6% to −11.8% after the five participants with TSH < 0.27 mIU/L were excluded. Among the 22 participants at or below the empirical 10th percentile, only 17 had fT4 available; among the five participants with TSH < 0.27 mIU/L, fT4 was available in four and was below the institutional interval in one. Selective testing and missing fT4 therefore preclude exclusion of overt or subclinical thyroid dysfunction in every low-TSH participant.

Most debate about age and TSH concerns the upper limit because that limit affects the classification of subclinical hypothyroidism [4,5,10]. The lower limit also has clinical relevance: persistent subclinical hyperthyroidism has been associated with coronary heart disease, atrial fibrillation, mortality [18], and fracture [19]. Low or inappropriately normal TSH may also occur with hypothalamic–pituitary dysfunction, for which interpretation requires concomitant fT4 and clinical assessment [1,2]. If a downward age shift at the lower end were reproduced in an appropriately selected reference population, it could influence biochemical classification. The present exploratory result does not establish such a shift or justify changing the lower limit.

Potential explanations include thyroid autonomy in an iodine-deficiency context, age-related hypothalamic–pituitary–thyroid regulation, unrecognized pituitary disease, referral and survivor effects, residual non-thyroidal illness, and chance. Iodine status among older adults in Ankara had been described as suboptimal more than a decade before this cohort [13], but urinary iodine, thyroid autoantibodies, systematic ultrasonography, and repeat TSH measurements were unavailable. Selective fT4 testing also prevents assessment of central thyroid dysfunction throughout the cohort. The data therefore cannot distinguish persistent physiology from transient suppression or selection effects, and the lower-tail finding should be considered hypothesis-generating only.

### 4.5. Strengths and Limitations

This study has several important strengths. To our knowledge, it is among the few studies from Türkiye to examine TSH in a geriatric cohort screened at the participant level, rather than through indirect laboratory-database methods. Eligibility was assessed through documented clinical history, physical examination, medication review, and laboratory screening; all eligible records from the predefined window were considered. Source-data validation recalculated age from dates, enforced the visit window, and resolved eligibility and denominator discrepancies before analysis. Continuous age modeling, HC3 robust standard errors, bootstrap quantile intervals, and sensitivity analyses, including adjustment for comorbidities and medication count and restriction by the institutional TSH and fT4 intervals, consistently showed no statistically significant age association. Female-sex point estimates were positive across models, although their precision varied in restricted analyses. All TSH measurements were performed on a single assay platform in one central laboratory, avoiding inter-assay variability.

This study has several limitations. First, the single-center, retrospective, cross-sectional design cannot separate aging from cohort, survivor, or referral effects, and only 17 participants were aged ≥85 years. Because the sample size was determined for the prevalence objective of the parent thesis, precision for the present objective was limited: the study could detect with 80% power a change of about 10% in TSH per five years of age and, for the ≥85-year group versus the 65–74-year group, only a difference of roughly 43% (decrease) or 77% (increase); the CIs in Table 2 and Table 3 should be read with these limits in mind. Second, TSH was measured once. Samples were drawn in the morning after an overnight fast, but the clock time of sampling and specimen handling were not documented, so diurnal and pre-analytical variation could not be assessed; although 91.2% of samples were drawn on the day of the visit, short-term biological variation, seasonality, and persistence of abnormalities could not be standardized. Third, thyroid disease was excluded on the basis of documented history, examination, medications, and available laboratory results; thyroid autoantibodies and urinary iodine were not measured, fT4 was available in 78.6% of participants, and thyroid ultrasonography was not systematically available. Undiagnosed or subclinical thyroid dysfunction therefore cannot be excluded, as illustrated by the three participants with TSH outside the interval and low fT4 (Supplementary Table S3); the sensitivity analyses excluding participants with TSH outside the interval or with suppressed TSH address this partially and showed attenuation of the age estimate. Fourth, 250 of the 456 screened patients were excluded, most often for elevated inflammatory markers or insufficient laboratory data, which may have introduced selection bias; individual-level characteristics of the excluded patients were not retained, so they could not be compared with those of the included cohort. Fifth, adjustment was limited to the comorbidities recorded in the electronic record and to medication count; residual confounding by unrecorded conditions, specific drugs, body mass index, iodine status, and thyroid autoimmunity is possible. Finally, missing data were minimal for comorbidity fields (one to three participants) but substantial for fT4 and fT3 because of selective testing, and all models used complete cases.

## 5. Conclusions

Among 206 clinically screened geriatric outpatients, TSH did not differ significantly across conventional age groups, and the adjusted continuous age association did not reach statistical significance. The primary analysis indicated higher TSH in women, with positive but variably precise estimates across sensitivity analyses. An exploratory negative age estimate at the lower end of the TSH distribution was not confirmed by formal tests of percentile differences and attenuated after exclusion of participants below the institutional lower limit; it requires independent replication. The findings support confirmation of isolated TSH abnormalities and consideration of within-person change and clinical context in older outpatients; whether sex-specific interpretation improves clinical classification cannot be determined from this cohort. Larger prospective multicenter studies with repeated, time-standardized TSH measurements, fT4 measurement in all participants, and assessment of thyroid autoantibodies and iodine status are needed.

## Figures and Tables

**Table 1:** Participant characteristics overall and by age group.

Characteristic	Overall (*n* = 206)	65–74 Years (*n* = 139)	75–84 Years (*n* = 50)	≥85 Years (*n* = 17)
Age, years, mean ± SD	73.9 ± 6.5	70.2 ± 2.8	79.5 ± 2.8	88.5 ± 2.8
Women, *n* (%)	122 (59.2)	87 (62.6)	23 (46.0)	12 (70.6)
TSH, mIU/L, median (IQR)	1.80 (1.25–2.61)	1.84 (1.32–2.69)	1.73 (1.14–2.36)	1.54 (1.05–1.95)
TSH in women, mIU/L, median (IQR) [*n*]	1.92 (1.47–2.70) [122]	1.97 (1.57–2.72) [87]	1.79 (1.16–2.73) [23]	1.41 (0.91–2.09) [12]
TSH in men, mIU/L, median (IQR) [*n*]	1.51 (1.00–2.20) [84]	1.46 (0.98–2.34) [52]	1.56 (1.01–2.03) [27]	1.62 (1.34–1.95) [5]
TSH < 0.27 mIU/L, *n* (%)	5 (2.4)	3 (2.2)	1 (2.0)	1 (5.9)
TSH > 4.20 mIU/L, *n* (%)	12 (5.8)	10 (7.2)	2 (4.0)	0 (0.0)
fT4 available, *n* (%)	162 (78.6)	115 (82.7)	36 (72.0)	11 (64.7)
Hypertension, *n*/*N* (%)	137/205 (66.8)	85/138 (61.6)	37/50 (74.0)	15/17 (88.2)
Diabetes mellitus, *n*/*N* (%)	82/205 (40.0)	52/138 (37.7)	26/50 (52.0)	4/17 (23.5)
Coronary artery disease, *n*/*N* (%)	61/205 (29.8)	37/138 (26.8)	18/50 (36.0)	6/17 (35.3)
Chronic kidney disease, *n*/*N* (%)	18/205 (8.8)	9/138 (6.5)	9/50 (18.0)	0/17 (0.0)
Dementia, *n*/*N* (%)	18/203 (8.9)	7/136 (5.1)	8/50 (16.0)	3/17 (17.6)
Regular medications, *n*, median (IQR)	3.5 (2.0–5.0)	3.0 (1.0–5.0)	4.5 (2.0–6.8)	4.0 (2.0–5.0)
≥5 regular medications, *n* (%)	76 (36.9)	44 (31.7)	25 (50.0)	7 (41.2)

Data are *n* (%) unless otherwise stated. Comorbidity fields were missing for one participant for each of hypertension, diabetes, coronary artery disease, and chronic kidney disease and for three participants for dementia; the extent of missing data for every variable is given in Supplementary Table S1. fT4 was requested selectively at the treating physician’s discretion. Regular medications were counted from the medication list recorded at the visit. fT4, free thyroxine; IQR, interquartile range; SD, standard deviation; TSH, thyroid-stimulating hormone.

**Table 2:** Association of age and sex with TSH: pre-specified analyses and sensitivity analyses.

Analysis	Contrast	Estimate	95% CI	*p* Value	*n*
**A. Pre-specified primary analysis**					
Robust log-linear model, adjusted for sex	Age, per 5 years	−6.6%	−13.1% to +0.5%	0.066	206
**B. Pre-specified secondary analyses**					
Spearman correlation	Age vs. TSH	ρ = −0.106	—	0.130	206
Kruskal–Wallis test	Three age groups	H = 3.78	—	0.151	206
Robust log-linear model, adjusted for sex ^a^	75–84 vs. 65–74 years	−6.4%	−26.6% to +19.4%	0.594	206
	≥85 vs. 65–74 years	−25.9%	−50.4% to +10.5%	0.140	206
Mann–Whitney U test	Women vs. men	Median 1.92 vs. 1.51 mIU/L	—	0.010	206
Robust log-linear model, adjusted for age (primary model)	Women vs. men	+25.5%	+1.3% to +55.5%	0.037	206
**C. Pre-specified sensitivity analysis**					
TSH within 0.27–4.20 mIU/L	Age, per 5 years	−3.2%	−8.6% to +2.6%	0.274	189
	Women vs. men	+23.2%	+5.7% to +43.6%	0.008	189
**D. Post hoc sensitivity analyses (added at revision) ^b^**					
Adjusted for sex and comorbidities ^c^	Age, per 5 years	−6.7%	−13.5% to +0.7%	0.077	203
	Women vs. men	+29.6%	+4.2% to +61.1%	0.020	203
Adjusted for sex, comorbidities, and number of regular medications ^c^	Age, per 5 years	−6.5%	−13.4% to +1.0%	0.086	203
	Women vs. men	+29.1%	+3.9% to +60.6%	0.022	203
Excluding TSH < 0.27 mIU/L	Age, per 5 years	−3.9%	−9.8% to +2.4%	0.221	201
	Women vs. men	+18.9%	+0.6% to +40.6%	0.043	201
Excluding TSH > 4.20 mIU/L	Age, per 5 years	−5.8%	−12.1% to +0.8%	0.084	194
	Women vs. men	+30.6%	+6.1% to +60.7%	0.012	194
TSH within 0.27–4.20 mIU/L and fT4 within 12–22 pmol/L ^d^	Age, per 5 years	−3.0%	−10.3% to +4.9%	0.445	142
	Women vs. men	+37.4%	+15.1% to +64.0%	0.001	142
Sex-stratified model, women	Age, per 5 years	−8.7%	−17.0% to +0.3%	0.059	122
Sex-stratified model, men	Age, per 5 years	−2.7%	−13.4% to +9.3%	0.643	84
Age-by-sex interaction	Age × sex	—	—	0.397	206

Regression estimates are percentage differences in TSH from models of log-transformed TSH with HC3 robust standard errors and t-based inference; age and sex were mutually adjusted in every regression model. *n* is the number of participants contributing to each analysis (complete cases). ^a^ Age was modeled as three categories (reference, 65–74 years) with adjustment for sex; pairwise non-parametric testing was not pursued because the omnibus test was not significant, and these contrasts are shown to convey precision. ^b^ Not pre-specified; added in response to peer review and not adjusted for multiplicity. ^c^ Comorbidities: hypertension, diabetes mellitus, coronary artery disease, chronic kidney disease, and dementia (variance inflation factors all <1.2); full coefficients in Supplementary Table S2. ^d^ Restricted to participants with fT4 available and within the laboratory interval. CI, confidence interval; fT4, free thyroxine; TSH, thyroid-stimulating hormone.

**Table 3:** Exploratory quantile regression of TSH on age and sex across the conditional distribution (*n* = 206).

**(A) Percentile-Specific Estimates**						
**TSH Percentile**	**Age Effect per 5 Years**	**95% Bootstrap CI**	**Bootstrap SE ^a^**	**Women vs. Men**	**95% Bootstrap CI**	**Bootstrap SE ^a^**
10th	−19.6%	−37.4% to −6.6%	0.107	+43.1%	+2.9% to +123.2%	0.197
25th ^b^	−8.7%	−18.2% to +1.3%	0.055	+42.8%	+11.6% to +78.5%	0.121
50th	−1.7%	−8.4% to +7.2%	0.036	+26.0%	+1.3% to +49.4%	0.102
75th ^b^	0.0%	−10.5% to +6.1%	0.044	+22.7%	−12.5% to +46.9%	0.136
90th	−5.8%	−13.9% to +4.7%	0.054	−1.2%	−25.7% to +29.4%	0.153
**(B) Differences Between Percentile-Specific Coefficients (Added at Revision)**						
**Comparison**		**Age: Difference (Log Units) ^c^**	**95% Bootstrap CI**	**Bootstrap *p* ^d^**	**Sex: Difference (Log Units) ^c^**	**95% Bootstrap CI (Bootstrap *p*) ^d^**
10th vs. 50th percentile		−0.20	−0.45 to −0.05	0.006 ^e^	+0.13	−0.17 to +0.59 (0.30)
10th vs. 90th percentile		−0.16	−0.44 to +0.01	0.076	+0.37	−0.03 to +0.92 (0.067)
50th vs. 90th percentile		+0.04	−0.08 to +0.14	0.48	+0.24	−0.06 to +0.53 (0.14)
Joint test of equality across the 10th, 50th, and 90th percentiles ^f^		χ^2^ = 4.09, df = 2	—	0.13	χ^2^ = 3.12, df = 2	— (0.21)

Estimates are percentage differences in TSH from exploratory quantile regressions of log-transformed TSH; age and sex were mutually adjusted. Percentile CIs were obtained from 5000 case-bootstrap resamples (seed 20260829), were not adjusted for multiplicity, and should be interpreted as hypothesis-generating. Twenty-two observations lay at or below the fitted 10th-percentile line. The 10th-percentile age estimate ranged from −21.7% to −19.6% when any single participant was deleted (leave-one-out) and was −11.8% per five years (95% bootstrap CI, −21.9% to −2.0%; *n* = 201) after exclusion of the five participants with TSH < 0.27 mIU/L. ^a^ Standard deviation of the bootstrap distribution of the coefficient, on the log scale. ^b^ The 25th and 75th percentiles were added for graphical display (Figure 2) and were not among the originally reported percentiles. ^c^ Difference between the two percentile-specific coefficients on the log(TSH) scale; a negative value for age indicates a more negative age coefficient at the lower percentile. ^d^ Two-sided *p* value from the paired bootstrap distribution of the difference, computed from the same resamples (2 × the smaller tail proportion). ^e^ A normal-approximation test using the bootstrap standard error gave *p* = 0.057 for this comparison; the discrepancy reflects skewness of the bootstrap distribution in the lower tail. ^f^ Wald test of equality of the age (or sex) coefficients across the three percentiles using the bootstrap covariance matrix. CI, confidence interval; df, degrees of freedom; SE, standard error; TSH, thyroid-stimulating hormone.

## Data Availability

De-identified participant-level data are available from the corresponding author upon reasonable request, subject to institutional and ethics approval. The data are not publicly available because they contain sensitive health information and are subject to confidentiality restrictions.

## Supplementary Materials

The following supporting information can be downloaded at https://www.mdpi.com/article/10.3390/jcm15208059/s1, Table S1: Missing data by variable and analytic sample for each model; Table S2: Covariate-adjusted robust log-linear models of TSH (percentage differences with 95% CIs); Table S3: Individual thyroid-function values of the 17 participants with TSH outside the institutional laboratory interval (0.27–4.20 mIU/L).

## References

[1] Taylor P.N., Lansdown A., Witczak J., Khan R., Rees A., Dayan C.M., Okosieme O. (2023). Age-related variation in thyroid function—A narrative review highlighting important implications for research and clinical practice. Thyroid Res..

[2] Jasim S., Papaleontiou M. (2025). Considerations in the diagnosis and management of thyroid dysfunction in older adults. Thyroid.

[3] Biondi B., Cappola A.R. (2022). Subclinical hypothyroidism in older individuals. Lancet Diabetes Endocrinol..

[4] Hollowell J.G., Staehling N.W., Flanders W.D., Hannon W.H., Gunter E.W., Spencer C.A., Braverman L.E. (2002). Serum TSH, T4, and thyroid antibodies in the United States population (1988 to 1994): National Health and Nutrition Examination Survey (NHANES III). J. Clin. Endocrinol. Metab..

[5] Surks M.I., Hollowell J.G. (2007). Age-specific distribution of serum thyrotropin and antithyroid antibodies in the US population: Implications for the prevalence of subclinical hypothyroidism. J. Clin. Endocrinol. Metab..

[6] Bremner A.P., Feddema P., Leedman P.J., Brown S.J., Beilby J.P., Lim E.M., Wilson S.G., O’Leary P.C., Walsh J.P. (2012). Age-related changes in thyroid function: A longitudinal study of a community-based cohort. J. Clin. Endocrinol. Metab..

[7] Xing D., Liu D., Li R., Zhou Q., Xu J. (2021). Factors influencing the reference interval of thyroid-stimulating hormone in healthy adults: A systematic review and meta-analysis. Clin. Endocrinol..

[8] Li Q., Tang Y., Yu X., Qin G., Tian L., Cheng L., Lu Y., Zhao Z., Liu L., Zhang K. (2025). Thyroid function reference intervals by age, sex, and race: A cross-sectional study. Ann. Intern. Med..

[9] Zhai X., Li Y., Teng X., Teng W., Shi X., Shan Z. (2025). Thyrotropin reference interval in older adults. Endocr. Connect..

[10] Raverot V., Bonjour M., Abeillon du Payrat J., Perrin P., Roucher-Boulez F., Lasolle H., Subtil F., Borson-Chazot F. (2020). Age- and sex-specific TSH upper-limit reference intervals in the general French population: There is a need to adjust our actual practices. J. Clin. Med..

[11] Çatak Z., Fener H., Ayyıldız H., Çokgüler Z.Ş. (2026). Establishing age- and sex-specific reference intervals for thyroid function tests in the older people of Eastern Anatolia: A population-based indirect approach. Medicina.

[12] Yildiz Z., Dağdelen L.K. (2023). Reference intervals for thyroid disorders calculated by indirect method and comparison with reference change values. Biochem. Med..

[13] Atmis V., Bülbül B., Bahşi R., Gümüşsoy M., Yalçin A., Doğan Z., Demir Ö., Erdoğan M., Atli T. (2021). Iodine concentration and prevalence of thyroid disease in older people after salt iodization in Turkey. East. Mediterr. Health J..

[14] von Elm E., Altman D.G., Egger M., Pocock S.J., Gøtzsche P.C., Vandenbroucke J.P., STROBE Initiative (2007). The Strengthening the Reporting of Observational Studies in Epidemiology (STROBE) statement: Guidelines for reporting observational studies. PLoS Med..

[15] Clinical and Laboratory Standards Institute (2010). Defining, Establishing, and Verifying Reference Intervals in the Clinical Laboratory.

[16] Kiren G., Yılmaz Çalık G., Güngören M., Çubukçu H.C. (2026). Age- and sex-specific reference intervals for TSH, FT4, and FT3 derived from the Turkish multi-center cohort. Diagnostics.

[17] Pearce S.H.S., Razvi S., Yadegarfar M.E., Martin-Ruiz C., Kingston A., Collerton J., Visser T.J., Kirkwood T.B., Jagger C. (2016). Serum thyroid function, mortality and disability in advanced old age: The Newcastle 85+ Study. J. Clin. Endocrinol. Metab..

[18] Collet T.H., Gussekloo J., Bauer D.C., den Elzen W.P.J., Cappola A.R., Balmer P., Iervasi G., Åsvold B.O., Sgarbi J.A., Völzke H. (2012). Subclinical hyperthyroidism and the risk of coronary heart disease and mortality. Arch. Intern. Med..

[19] Blum M.R., Bauer D.C., Collet T.H., Fink H.A., Cappola A.R., da Costa B.R., Wirth C.D., Peeters R.P., Åsvold B.O., den Elzen W.P.J. (2015). Subclinical thyroid dysfunction and fracture risk: A meta-analysis. JAMA.

