# Supplementary material for: Serum Thyrotropin Levels Across Older Age Groups in Geriatric Outpatients Without Documented or Clinically Evident Thyroid Disease: A Retrospective Cross-Sectional Study
Source: J Clin Med. 2026 Oct 17;15(20):8059. doi: 10.3390/jcm15208059 (PMC13705884; doi:10.3390/jcm15208059)
Supplement: Supplementary file 1 [file jcm-15-08059-s001.zip › jcm-4578902-supplementary.pdf]

Supplementary Materials

Serum Thyrotropin Levels across Older Age Groups in Geriatric Outpatients without Documented or Clinically Evident Thyroid Disease: A Retrospective Cross-Sectional Study (jcm-4578902)

Table S1. Missing data by variable and analytic sample for each model.

Variable	Missing, n (%) of 206	Reason	Analyses affected
Age, sex, TSH	0 (0.0)	—	None
Hypertension	1 (0.5)	Comorbidity field not completed	Table 1 denominator; comorbidity-adjusted models
Diabetes mellitus	1 (0.5)	Comorbidity field not completed	As above
Coronary artery disease	1 (0.5)	Comorbidity field not completed	As above
Chronic kidney disease	1 (0.5)	Comorbidity field not completed	Table 1 denominator; CKD- and comorbidity-adjusted models
Dementia	3 (1.5)	Comorbidity field not completed	Table 1 denominator; comorbidity-adjusted models
Hyperlipidemia / depression / asthma / COPD	1 (0.5) / 4 (1.9) / 2 (1.0) / 0 (0.0)	Comorbidity field not completed	Not used in any analysis
Estimated GFR	2 (1.0)	Creatinine not reported	eGFR-adjusted model (Table S2) only
fT4	44 (21.4)	Requested selectively at physician's discretion	Descriptive fT4 statistics; sensitivity model requiring TSH and fT4 within institutional intervals (n = 142)
fT3	122 (59.2)	Requested selectively at physician's discretion	Descriptive fT3 statistics only
Laboratory sampling date	1 (0.5)	Date not recorded	Description of sampling timing only
Medication list	0 (0.0)	—	Medication-count model

Analytic sample by model (complete cases): primary model and pre-specified non-parametric tests, n = 206; institutional TSH-range sensitivity analysis, n = 189; comorbidity-adjusted models, n = 203; CKD-adjusted model, n = 205; eGFR-adjusted model, n = 201; model excluding TSH <0.27 mIU/L, n = 201; model excluding TSH >4.20 mIU/L, n = 194; sensitivity subset with TSH and fT4 within their institutional intervals, n = 142; sex-stratified models, n = 122 (women) and 84 (men); quantile regressions, n = 206. Missing values were not imputed. CKD, chronic kidney disease; COPD, chronic obstructive pulmonary disease; eGFR, estimated glomerular filtration rate; fT3, free triiodothyronine; fT4, free thyroxine; TSH, thyroid-stimulating hormone.

Table S2. Covariate-adjusted robust log-linear models of TSH (percentage differences with 95% CIs).

Term	Model 2: sex + comorbidities (n = 203)	Model 3: Model 2 + number of regular medications (n = 203)	CKD-only model (n = 205)	eGFR model [a] (n = 201)
Age, per 5 years	−6.7% (−13.5 to +0.7); p = 0.077	−6.5% (−13.4 to +1.0); p = 0.086	−6.0% (−12.5 to +1.1); p = 0.094	−3.6% (−11.1 to +4.5); p = 0.366
Women vs. men	+29.6% (+4.2 to +61.1); p = 0.020	+29.1% (+3.9 to +60.6); p = 0.022	+27.2% (+3.8 to +55.8); p = 0.020	+27.9% (+1.7 to +60.9); p = 0.035

Term	Model 2: sex + comorbidities (n = 203)	Model 3: Model 2 + number of regular medications (n = 203)	CKD-only model (n = 205)	eGFR model [a] (n = 201)
Hypertension	+10.6% (−11.7 to +38.5); p = 0.378	+14.8% (−9.0 to +45.0); p = 0.243	—	+16.0% (−8.1 to +46.3); p = 0.210
Diabetes mellitus	−2.0% (−18.9 to +18.5); p = 0.837	+3.9% (−14.4 to +26.0); p = 0.699	—	−0.4% (−19.2 to +22.9); p = 0.972
Coronary artery disease	+11.2% (−11.0 to +38.9); p = 0.348	+18.7% (−6.9 to +51.3); p = 0.166	—	+8.3% (−13.5 to +35.5); p = 0.486
Chronic kidney disease	−4.0% (−42.6 to +60.3); p = 0.874	−0.5% (−39.4 to +63.5); p = 0.985	−4.1% (−43.8 to +63.5); p = 0.876	—
eGFR, per 1 mL/min/1.73 m^2	—	—	—	+0.5% (−0.1 to +1.1); p = 0.103
Dementia	−0.8% (−25.1 to +31.6); p = 0.958	+3.9% (−22.1 to +38.5); p = 0.795	—	−2.0% (−25.8 to +29.4); p = 0.885
Regular medications, per additional drug	—	−3.0% (−6.9 to +1.0); p = 0.139	—	—

All models use log-transformed TSH with HC3 robust standard errors and t-based inference. Variance inflation factors in Model 2 were all <1.2. [a] Shown for completeness only: creatinine-based eGFR equations include age as an input, so adjusting for eGFR partly adjusts for age itself and attenuates the age coefficient by construction; the binary chronic kidney disease diagnosis was therefore retained in the main sensitivity models. CI, confidence interval; CKD, chronic kidney disease; eGFR, estimated glomerular filtration rate; TSH, thyroid-stimulating hormone.

Table S3. Individual thyroid-function values of the 17 participants with TSH outside the institutional laboratory interval (0.27–4.20 mIU/L).

No.	Age group, years	Sex	TSH, mIU/L	fT4, pmol/L	fT3, pmol/L
1	65–74	Man	0.05	14.1	5.64
2	65–74	Man	0.06	18.0	—
3	65–74	Woman	0.07	11.3 [a]	4.88
4	≥85	Woman	0.17	14.3	—
5	75–84	Man	0.17	not requested	—
6	65–74	Man	4.27	12.7	—
7	65–74	Man	4.39	18.7	4.45
8	65–74	Woman	4.45	16.8	—
9	65–74	Woman	4.70	14.3	—
10	65–74	Man	4.70	not requested	—
11	65–74	Woman	4.79	16.3	—
12	65–74	Man	5.09	15.4	—
13	65–74	Woman	5.17	13.7	4.50
14	65–74	Woman	5.45	18.4	4.45
15	65–74	Man	5.77	13.8	4.60
16	75–84	Man	5.97	11.5 [a]	4.14
17	75–84	Woman	11.30	11.8 [a]	—

Participants are ordered by TSH. fT4 and fT3 were requested selectively; “not requested” or “—” indicates that a result was unavailable. Institutional laboratory reference intervals: TSH 0.27–4.20 mIU/L; fT4 12–22 pmol/L. a fT4 below the laboratory interval. Three of the 15 tested participants had fT4 below the interval; none had an elevated fT4. fT3, free triiodothyronine; fT4, free thyroxine; TSH, thyroid-stimulating hormone.
